# Psychometric Validation of the Iowa Infant Feeding Attitude Scale Among Healthcare Students in Vietnam

**DOI:** 10.3390/healthcare13111233

**Published:** 2025-05-23

**Authors:** My Thi Thuy Dang, Marianne Lin-Lewry, Cai Thi Thuy Nguyen, Gabrielle T. Lee, Su-Ru Chen, Shu-Yu Kuo

**Affiliations:** 1 Faculty of Nursing, College of Medicine and Pharmacy, Tra Vinh University, Tra Vinh City 87000, Vietnam; dttmy@tvu.edu.vn; 2 School of Nursing, College of Nursing, Taipei Medical University, 250 Wuxing St., Taipei 110, Taiwan; marianne01011414@gmail.com; 3 Faculty of Nursing and Midwifery, Hanoi Medical University, Hanoi 100000, Vietnam; nguyenthithuycai@gmail.com; 4 Research Institute for the Humanities and Social Sciences, National Science and Technology Council, Taipei 100029, Taiwan; 5 Center for the Advancement of the Humanities and Social Sciences, National Taiwan University, Taipei 100029, Taiwan; 6 Applied Psychology, Faculty of Education, Western University, London, ON N6A 3K7, Canada; gabtsai@hotmail.com; 7 Post-Baccalaureate Program in Nursing, College of Nursing, Taipei Medical University, Taipei 110, Taiwan; suru@tmu.edu.tw; 8 Nursing Department, Taipei Medical University Hospital, Taipei 110, Taiwan

**Keywords:** breastfeeding, healthcare, student, attitude, validity, reliability

## Abstract

**Background:** The Iowa Infant Feeding Attitude Scale (IIFAS) is widely used to assess breastfeeding attitudes, which are linked to positive breastfeeding practices. However, its psychometric validation in Southeast Asian healthcare students remains limited. **Objectives:** To investigate the reliability and validity of the IIFAS among Vietnamese healthcare students. **Methods:** A cross-sectional study was conducted at three medical universities in Vietnam. A total of 542 healthcare students, including medical, nursing, and midwifery students, participated. The students completed the Iowa Infant Feeding Attitude Scale, Breastfeeding Knowledge Scale, and Generalized Anxiety Disorder Scale. The reliability was assessed through the internal consistency and test–retest reliability. The construct validity was tested using exploratory and confirmatory factor analysis. The divergent validity, convergent validity, and known-group comparison were also assessed. **Results:** The IIFAS showed an excellent internal consistency (Cronbach’s α = 0.94) and test–retest reliability (intraclass correlation = 0.91). A two-factor structure of the Vietnamese IIFAS was identified using exploratory and confirmatory factor analysis with satisfactory fit indices (χ^2^/df = 1.318, comparative fit index = 0.985, Tucker–Lewis Index = 0.983, and Root Mean Square Error of Approximation = 0.034). Breastfeeding attitudes positively correlated with breastfeeding knowledge (r = 0.74, *p* < 0.001) and negatively correlated with anxiety symptoms (r = −0.13, *p* = 0.04). Students who were older, in a higher academic year, and majoring in medicine had significantly higher breastfeeding attitude scores (ps < 0.05). **Conclusions:** The Vietnamese version of the IIFAS demonstrates excellent reliability and validity, making it a robust tool for assessing breastfeeding attitudes and informing tailored educational programs among healthcare students.

## 1. Introduction

Breastfeeding support from healthcare professionals is essential for helping mothers initiate and sustain exclusive breastfeeding practices [1]. To ensure that future professionals are adequately prepared, incorporating breastfeeding courses into the educational curricula of medical, midwifery, and nursing students has been recommended [2]. In Vietnam, breastfeeding practice is recognized as a key public health priority. The Centers of Excellence for Breastfeeding model has been established to implement the Baby-Friendly Hospital Initiative and to support mothers in initiating early breastfeeding and sustaining exclusive breastfeeding [3]. However, only 23.5% of Vietnamese mothers initiate breastfeeding within one hour after birth, and 45.4% practice exclusive breastfeeding for infants aged 0 to 6 months [4]. Recent research has suggested that enhancing education and support from healthcare professionals is critical for improving breastfeeding outcomes [5]. To prepare the healthcare students in the core breastfeeding competencies is a vital component of national efforts in promoting breastfeeding practices [6]. Nevertheless, a systematic review showed that healthcare students had only moderate breastfeeding attitudes, suggesting the importance of assessing students’ attitudes promptly to better breastfeeding education outcomes [7]. Hence, establishing a validated breastfeeding attitude tool among healthcare students is crucial for optimal breastfeeding practice.

The Iowa Infant Feeding Attitude Scale (IIFAS) was originally developed to assess breastfeeding attitudes among postnatal women and to serve as a screening instrument [8]. The validation and reliability of this scale have been proven in various populations of mothers and fathers from European, Chinese, and Latin American countries [9,10,11]. However, research has shown that breastfeeding mothers from different cultures have differing IIFAS factor structures. For example, in Germany, a two-factor model was identified [9], consistent with the dimensions reported in the original questionnaire [8]. In contrast, in Asia, studies have reported a three-factor structure in Singapore [12] and a four-factor structure in China [11]. Recently, the IIFAS has been increasingly applied to college students in countries such as China [13] and Egypt [14] as part of efforts to promote breastfeeding education. Despite the growing body of research including student populations, the psychometric properties of the IIFAS have not been examined among healthcare students in the Vietnamese context. This literature gap highlights the need to investigate the validity and reliability of IIFAS in Vietnamese healthcare students.

Several demographic and school characteristics have been found to be associated with healthcare students’ attitudes toward breastfeeding, including the student’s age, gender, study major, and academic year [7,15]. Notably, medical students and those in more advanced academic years tend to report positive attitudes toward breastfeeding [7]. Additionally, a positive correlation has been observed between breastfeeding knowledge and favorable attitudes among healthcare students [16]. While most studies on mothers have found that high levels of anxiety negatively impact breastfeeding attitudes, the association between anxiety symptoms and breastfeeding attitude in healthcare students remains limited known. Previous research suggests that individuals with higher levels of anxiety symptoms often develop negative expectations when faced with unfamiliar or challenging situations [17,18]. For healthcare students, learning to supporting breastfeeding is an expected professional competency. However, students experiencing higher anxiety may perceive breastfeeding education as an unfamiliar or demanding task, which could negatively influence their attitudes toward breastfeeding. Therefore, further investigation is needed to better understand the relationship between anxiety symptoms and breastfeeding attitudes in a student population.

The objectives of this study were to evaluate the reliability and validity of the IIFAS among healthcare students in Vietnam, including its construct validity, convergent, divergent validity, and known-group validity.

## 2. Methods

### 2.1. Study Design

A cross-sectional study design was implemented among healthcare students at three medical universities in Southern Vietnam between October 2023 and February 2024. These institutions provide breastfeeding-related education through both lectures and clinical practicums. The study was approved by the Institutional Review Board of Taipei Medical University in Taiwan, approval code N202308018, and the Hanoi University of Public Health in Vietnam, approval code 394/2023/YTCC-HD3. The study’s purpose and data collection procedures were explained to participants, and written informed consent was obtained before their participation.

### 2.2. Participants

A convenience sampling method was used to recruit undergraduate students enrolled in medicine, nursing, and midwifery programs. The recruitment flyer was distributed to all students in these programs, and eligible students were invited to participate voluntarily. The inclusion criteria were as follows: (1) age of 18 years or older and (2) completion of clinical training related to breastfeeding as part of their respective program (e.g., third- or fourth-year nursing and midwifery students; fifth-year medical students). Students who were not willing to participate were excluded. The sample size was determined based on the guideline recommending at least 10 participants per item for factor analysis [19]. For the 17-item IIFAS, a minimum of 170 participants was required for each of the exploratory factor analysis (EFA) and confirmatory factor analysis (CFA) phases. EFA and CFA were conducted using separate, randomly assigned samples to achieve independent analyses. Accordingly, a total minimum sample size of 340 was required. The target sample size was estimated as at least 374 participants after taking account for a potential 10% nonresponse or incompletion rate.

### 2.3. Translation Process of the Iowa Infant Feeding Attitude Scale (IIFAS)

The original IIFAS was translated into Vietnamese with written permission from the author who developed the tool [8]. The translation process followed standard forward and back translation guidelines [19]. First, two bilingual translators who were native Vietnamese speakers fluent in English independently translated the scale for the forward translation process. An independent researcher then reviewed the translated versions to identify any inconsistencies and generated a preliminary Vietnamese version of the tool. Next, this version was back-translated into English by two bilingual translators who were unfamiliar with the original scale. Both the forward- and back-translated versions were subsequently reviewed by an expert panel comprising six professionals in maternal and child health to ensure language equivalence and cultural relevance. The expert panel discussed cultural context and clarified the ambiguous terms to confirm the scale’s relevance and appropriateness for the Vietnamese university students.

Content validity was evaluated and obtained based on the results of a panel of six experts. The experts were selected based on their professional qualifications and more than 10 years of experience in maternal and infant healthcare. The panel comprised pediatric nursing lecturers (n = 2), maternity nurses (n = 2), and midwives (n = 2), all with expertise relevant to breastfeeding practice. The experts rated all items to evaluate clarity and relevance using a four-point Likert (1 = not clear/relevant and 4 = highly clear/relevant). The item-level content validity index (I-CVI) > 0.80 and the scale-level content validity index (S-CVI) > 0.90 are used as psychometric satisfaction levels to determine the content validity [20]. The I-CVI in our study ranged from 0.83 to 1.00 for clarity and relevance, and the S-CVI was 0.97 for clarity and 0.99 for relevance.

A pilot test with 30 healthcare students was conducted to evaluate the clarity and comprehension of the Vietnamese IIFAS. Students reported that the tool was easy to understand and appropriate for evaluating breastfeeding attitudes. No further modifications were needed.

### 2.4. Measurement

#### 2.4.1. The Iowa Infant Feeding Attitude Scale (IIFAS)

The Vietnamese version of the IIFAS was used to measure attitudes toward breastfeeding. The 17-item scale is rated on a five-point Likert scale, ranging from 1 (strongly disagree) to 5 (strongly agree), with total scores ranging from 17 to 85. Higher scores indicate more positive attitudes toward breastfeeding. The original scale demonstrated good internal consistency, with a Cronbach’s α of 0.86 [8].

#### 2.4.2. Breastfeeding Knowledge

To evaluate healthcare students’ breastfeeding knowledge, our research team developed a 17-item True/False questionnaire adapted from existing instruments for the Vietnamese context [21,22,23]. This self-report measures the four domains of breastfeeding knowledge: the physiology of breastfeeding (3 items), breastfeeding adequacy (3 items), common breastfeeding problems (6 items), and breastfeeding management (5 items). Each correct answer scored 1 point; incorrect or blank responses scored 0. The total score ranged from 0 to 17, with higher scores reflecting higher breastfeeding knowledge level. The breastfeeding knowledge tool achieved an I-CVI score of 1.00 for clarity and 0.83 to 1.00 for relevance. The S-CVI also had high scores (1.00 for clarity and 0.98 for relevance). The internal consistency was assessed using the Kuder–Richardson 20 (KR-20) test, yielding a reliability coefficient of 0.79, indicating acceptable reliability.

#### 2.4.3. Generalized Anxiety Disorder Scale (GAD-7)

The Vietnamese version of Generalized Anxiety Disorder (GAD-7) scale is used for assessing anxiety symptoms over the past two weeks [24]. The 7-item scale used a 4-point Likert scale (0 = not at all, 3 = nearly every day), yielding total scores ranging from 0 to 21, with higher scores indicating increased anxiety levels. The GAD-7 demonstrated excellent reliability among Vietnamese students (Cronbach’s α = 0.94) [24]. In this study, Cronbach’s alpha was 0.87, indicating good internal consistency.

#### 2.4.4. Demographic and Academic Variables

Demographic and academic characteristics, including age, gender, grade point average, religion, ethnicity, academic year, study major, and previous breastfeeding support experience, were assessed based on prior studies [25,26].

### 2.5. Data Collection Procedures

The questionnaires were administered at the start of class sessions. To assess test–retest reliability, follow-up data were collected from 30 randomly selected students two weeks later.

### 2.6. Data Analysis

Data were analyzed using the Statistical Package for the Social Science (SPSS) version 25, IBM SPSS AMOS version 24, and R software version 4.5.0. Descriptive statistics were used to summarize categorical variables (frequencies and percentages) and continuous variables (means and standard deviations). The normality of variable distributions was assessed using skewness (ranging from −2 to +2) and kurtosis (ranging from −10 to +10) index for normality of variables [27]. To evaluate reliability, internal consistency was assessed using Cronbach’s alpha (≥0.70) for IIFAS [28] and Kuder–Richardson 20 (KR-20) (≥0.70) for the breastfeeding knowledge scale [29]. Test–retest reliability was examined using the intraclass correlation coefficient (ICC), with a value greater than 0.9 indicating excellent reliability [30].

Construct validity was assessed using exploratory factor analysis (EFA) and confirmatory factor analysis (CFA). The adequacy of the sample for EFA was evaluated using the Kaiser–Meyer–Olkin (KMO) measure (>0.60) [31] and Bartlett’s test of sphericity (*p* < 0.05) [32]. The minimum residual (MINERS) extraction method with oblimin rotation was adopted to identify the underlying factor structure while accounting for both common and unique variance among the items [33]. The number of factors was determined based on eigenvalues greater than 1.0 [20], inspection of the scree plot [34], and factor loadings ≥ 0.40 [20].

CFA was conducted to confirm the factor structure identified in EFA using maximum likelihood estimation. Model fit was evaluated with Chi-squared/degrees of freedom (χ^2^/df < 3), goodness-of-fit index (GFI > 0.90), adjusted goodness-of-fit index (AGFI > 0.90), Tucker–Lewis Index (TLI > 0.90), comparative fit index (CFI > 0.90), and Root Mean Square Error of Approximation (RMSEA < 0.08) [35]. The Akaike information criterion (AIC) was also considered, with lower values indicating better model fit [36].

Convergent validity was assessed using Pearson’s correlation coefficients between breastfeeding attitudes with knowledge scores. Divergent validity was examined using correlation index between the breastfeeding attitude score and anxiety symptom scores, with the hypothesis that better breastfeeding attitudes would be negatively associated with levels of anxiety. Known-group comparison was performed using the independent *t*-test and one-way ANOVA to examine differences in breastfeeding attitudes across demographic and academic characteristics. Measurement invariance across groups was examined following the procedures outlined by Millsap and Bornstein [37,38]. A four-step approach was applied, including the evaluation of configural, metric, scalar, and residual invariance [39,40]. Invariance was assessed using changes in the Tucker–Lewis Index (TLI) and Root Mean Square Error of Approximation (RMSEA), with ΔTLI values ≤ 0.01 and ΔRMSEA values ≤ 0.015 considered indicative of acceptable model fit. A *p*-value of < 0.05 was considered statistically significant.

## 3. Results

### 3.1. Characteristics of Participants

In this study, 542 healthcare students were included and randomly divided into two groups for an EFA sample (n = 271) and a CFA sample (n = 271) (Table 1). The mean age was 21.0 years (standard deviation, SD = 1.19), and the mean GPA was 2.93 (SD = 0.33). Most participants were female (73.8%), reported no religion (72.3%), and identified as Kinh ethnicity (85.2%). Most were in their fourth or fifth year of study. Nursing students comprised nearly half of the sample, and 81.9% reported prior experience assisting mothers with breastfeeding. The EFA and CFA samples were homogeneous, with no significant differences between groups (ps > 0.05).

### 3.2. Internal Consistency and Test–Retest Reliability of the IIFAS

The IIFAS had a mean total score of 67.5 (SD = 12.9), with item mean scores ranging from 3.90 (SD = 1.06) to 4.04 (SD = 1.11) (Table 2). The data demonstrated a normal distribution, with skewness values ranging from −1.28 to −0.94 and kurtosis values from 0.38 to 1.06. The “Alpha if the item deleted” values were consistently 0.93 across all items, indicating that each item contributed to the overall reliability and was consistent with the underlying construct. The Cronbach’s alpha reliability coefficient was 0.94 for the total IIFAS. The test–retest reliability of the IIFAS was excellent, with an intraclass correlation coefficient (ICC) of 0.91, further supporting the scale’s reliability.

### 3.3. Construct Validity

#### 3.3.1. Exploratory Factor Analysis (EFA)

To assess sampling adequacy for EFA, the Kaiser–Meyer–Olkin (KMO) measure of 0.97 indicated excellent sampling adequacy. Bartlett’s test of sphericity (χ^2^ = 5032.266, *p* < 0.001) confirmed the suitability of the data for factor analysis. The minimum residual (MINERS) extraction method with oblimin rotation identified two distinct factors with eigenvalues greater than 1.0, collectively explaining 54.0% of the total variance. The scree plot further supported this two-factor solution.

The first factor included nine items (items 3, 5, 7, 9, 12, 13, 15, 16, and 17) and was named “Favorable to Breastfeeding” with factor loadings ranging from 0.63 to 0.80. The second factor consisted of eight items (items 1, 2, 4, 6, 8, 10, 11, and 14) and was named ‘Favorable to Formula Feeding,’ with factor loadings ranging from 0.64 to 0.81 (Table 3).

#### 3.3.2. Confirmatory Factor Analysis (CFA)

**Figure 1:**
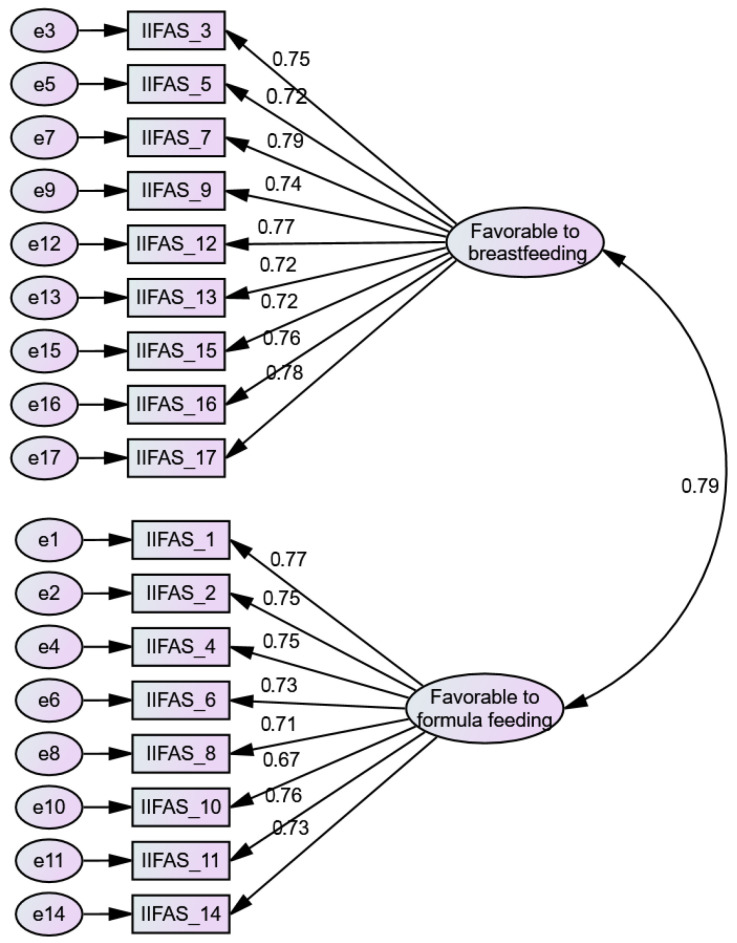
Confirmatory Factor Analysis of the Iowa Infant Feeding Attitude Scale among healthcare students in Vietnam.

The CFA results confirmed that the 17-item, two-factor model provided a good model fit. Fit indices were as follows: χ^2^/df = 1.318, GFI = 0.937, AGFI = 0.918, TLI = 0.983, CFI = 0.985, RMSEA = 0.034, and AIC = 225.572. Factor loading ranged from 0.67 to 0.79, indicating strong item-factor relationships (Supplementary Table S1). Specifically, items related to favorable attitudes toward breastfeeding (e.g., items 3, 5, 7, 9, 12, 13, 15, 16, and 17) loaded strongly on the first factor, with loadings between 0.72 and 0.79. Meanwhile, items reflecting favorable attitudes toward formula feeding (e.g., items 1, 2, 4, 6, 8, 10, 11, and 14) loaded onto the second factor, with loadings ranging from 0.67 to 0.77. The two latent factors were strongly correlated (r = 0.79), suggesting they capture related but distinct constructs. These findings support the two-factor structure identified in the CFA and confirm the scale’s robustness in measuring attitudes toward breastfeeding and formula feeding (Figure 1).

### 3.4. Convergent Validity

The Pearson correlation analysis examined the relationship between the IIFAS scores and breastfeeding knowledge. The results revealed that breastfeeding knowledge was strongly and positively correlated with the IIFAS total score (r = 0.74, *p* < 0.001), as well as with both the “Favorable to Breastfeeding” (r = 0.64, *p* < 0.001) and “Favorable to Formula Feeding” (r = 0.67, *p* < 0.001) subscales. The results showed that students with more positive attitudes toward breastfeeding tended to have greater breastfeeding knowledge (Table 4).

### 3.5. Divergent Validity

A significant but weak negative correlation was found between breastfeeding attitudes and anxiety symptoms (r = −0.13, *p* = 0.04). Similarly, the “Favorable to Breastfeeding” subscale was negatively correlated with anxiety symptoms (r = −0.16, *p* < 0.01), while no significant correlation was observed between anxiety symptoms and the “Favorable to Formula Feeding” subscale (r = −0.06, *p* > 0.05). This result supports the divergent validity of the Vietnamese version of the IIFAS, indicating that while the two constructs are related, they are conceptually distinct (Table 4).

### 3.6. Known-Group Comparison

The known-group analysis showed that the medical students had significantly more positive attitudes toward breastfeeding compared to the nursing (M = 65.80, SD = 14.39) and midwifery (M = 62.56, SD = 16.45) students (F = 5.68, *p* = 0.004), with the highest mean score observed among the medical students (M = 68.90, SD = 10.68). The differences between the nursing and midwife students were not significant. Age was positively associated with the breastfeeding attitude scores (r = 0.09, *p* = 0.04). The fifth-year students (M = 68.90, SD = 10.68) demonstrated significantly higher mean scores for breastfeeding attitudes than the fourth-year students (M = 65.35, SD = 14.85) (*p* = *0*.01). However, there were no significant differences between the third-year students and either the fourth-year or fifth-year students. No significant differences were observed based on gender (t = 0.74, *p* = 0.46), religion (t = 1.81, *p* = 0.07), ethnicity (t = 0.17, *p* = 0.87), or prior experience assisting with breastfeeding (t = −1.11, *p* = 0.27) (Table 5).

### 3.7. Measurement Invariance

Measurement invariance across the gender groups was supported through the multi-group CFA. Configural, metric (ΔTLI = 0.002, ΔRMSEA = 0.001), scalar (ΔTLI = 0.003, ΔRMSEA = 0.001), and strict invariance (ΔTLI = –0.003, ΔRMSEA = −0.003) were sequentially established, indicating that latent scores are comparable between the male and female participants.

## 4. Discussion

This study provides the first evidence of the psychometric properties of the Iowa Infant Feeding Attitude Scale (IIFAS) for assessing breastfeeding attitudes among healthcare students in Vietnam. Both the EFA and CFA confirmed a robust two-factor structure (“Favorable to Breastfeeding” and “Favorable to Formula Feeding”) with excellent internal consistency (Cronbach’s alpha = 0.94) and test–retest reliability (ICC = 0.91). Significant associations were identified between the breastfeeding attitudes and both breastfeeding knowledge and anxiety symptoms. A more positive breastfeeding attitude was found among the medical students, older students, and those in higher academic years. The Vietnamese version of the IIFAS is a valid and reliable tool for assessing breastfeeding attitudes among healthcare students, indicating its use in breastfeeding education efforts.

The IIFAS revealed excellent reliability by exceeding the recommended threshold of 0.70 [28]. The Cronbach’s alpha of the IIFAS was high, consistent with findings from previous studies among mothers in the United States [8], Germany [9], and Iran [41]. Furthermore, the scale exhibited high test–retest reliability, which was found to be similar in Iran [41] and Chinese mothers [11]. This demonstrated the strong temporal stability of the scale across diverse populations as well as mothers and students from different cultural populations. These results emphasize the reliability of the IIFAS and cultural adaptability in diverse cultural settings.

The results of the EFA and CFA confirmed a two-factor structure of the IIFAS, i.e., “Favorable to Breastfeeding” and “Favorable to Formula Feeding”. The two latent factors in the IIFAS model were strongly correlated (r = 0.79), suggesting that they may represent two closely related aspects of a broader, unified construct rather than distinct factors. This raises the possibility that the factors might be better represented as components of a single general attitude toward infant feeding with specific nuances. These two dimensions are consistent with the theoretical framework of the IIFAS, in which attitudes toward breastfeeding and formula feeding are theorized to be a continuum [8]. The two-factor structure observed in this study is consistent with previous studies among mothers in America [8], Germany [9], and Iran [41]. However, in contrast, a study conducted among pregnant women in Singapore identified a three-factor structure: “Favorable to Breastfeeding”, “Favorable to Formula Feeding” and “Convenience” [12]. In that study, the “Convenience” factor reflected attitudes related to the perceived ease or practicality of feeding methods, which may be influenced by cultural expectations, lifestyle, and work-related considerations. These differences in factor structures may be attributed to variations in sample characteristics (e.g., healthcare students vs. pregnant women), cultural norms, and different perceptions of breastfeeding within healthcare systems. Despite these variations, the consistent findings of a two-factor structure across studies suggest that the IIFAS captures core constructs related to infant feeding attitudes in different cultural regions. The IIFAS is a valuable tool for education to assess healthcare students’ attitudes toward breastfeeding. The use of the IIFAS in educational settings helps ensure that students are adequately prepared during both pre- and post-clinical practicums to effectively support breastfeeding among mothers in clinical practice.

The positive correlation between breastfeeding knowledge and attitudes is similar to the findings in existing studies that emphasize the close link between knowledge and attitudes [7]. The results highlight the importance of designing comprehensive breastfeeding education into the healthcare training curriculum to cultivate positive attitudes in future healthcare professionals. Such efforts could enhance their ability to provide effective breastfeeding support and improve outcomes for breastfeeding mothers.

The divergent validity analysis showed a significant negative correlation between breastfeeding attitudes, “Favorable to Breastfeeding” factor, and anxiety symptoms among healthcare students, consistent with previous findings on maternal breastfeeding attitudes [42]. Students experiencing high levels of anxiety may influence their breastfeeding-related activities, which, in turn, can change their attitude toward breastfeeding [43]. Past studies have shown that psychological techniques, including anxiety regulation strategies, are effective in reducing anxiety among healthcare students and could be integrated into educational programs [44]. Future research is warranted to explore whether incorporating stress management strategies into breastfeeding education programs can help reduce anxiety-related barriers and promote more positive attitudes toward breastfeeding among healthcare students.

In this study, the students in higher academic years demonstrated significantly more positive attitudes toward breastfeeding. Notably, the final-year students exhibited more positive attitudes due to extensive exposure to both theoretical knowledge and clinical training [7]. Clinical training environments allow students to apply their knowledge and develop supportive behaviors in real-world scenarios, which helps them engage with professional roles later [45]. Breastfeeding attitudes among medical students were also reported to be higher than those of their peers in nursing and midwifery. This may reflect differences in their curriculum content and professional roles related to breastfeeding support in Vietnam. In the final year of training, medical education programs focus on maternal and child health, preventive care, and breastfeeding’s role in reducing infant mortality in order to promote positive attitudes toward breastfeeding [46]. The competency of medical students in supporting breastfeeding can be acquired through various tasks such as counseling mothers, diagnosing lactation issues, and prescribing treatments. These findings highlight the need for comprehensive education across all healthcare disciplines to enhance breastfeeding support.

The results of the measurement invariance analysis indicated that the factor structure of the IIFAS was invariant across genders, which may help explain the lack of significant gender differences in breastfeeding attitude scores. This suggests that the IIFAS assesses breastfeeding attitudes consistently among both male and female students. Additionally, the absence of gender differences may be attributed to the fact that the roles of both male and female students in breastfeeding support are equally emphasized and encouraged within the breastfeeding curriculum [47]. The stability of the factor structure across gender groups further supports the validity and reliability of the IIFAS in assessing breastfeeding attitudes among student populations.

Several limitations should be acknowledged when interpreting the study results. First, the sample consisted of students from three universities in southern Vietnam, which may limit the generalizability of the results to healthcare students in other regions of the country. Future research should include students from universities across different regions. Second, this study used a cross-sectional design and included only students who had completed their clinical practicum; thus, changes in breastfeeding attitudes over time were not assessed. Longitudinal studies are needed to examine the patterns of change in attitudes throughout healthcare training and after graduation. Third, the sample was specific to healthcare students, with midwifery students (n = 34) being underrepresented compared to medical and nursing students. Future research should explore strategies to increase their participation, given their vital role as future breastfeeding professionals. In addition, applying the IIFAS to other populations in Vietnam, such as mothers or caregivers, is warranted to further validate its use. Finally, the breastfeeding attitude was assessed using a self-reported instrument, which may be subject to social desirability bias and should be interpreted with caution.

### Implications

This study offers both theoretical and practical implications for healthcare education in Vietnam. The validated Vietnamese version of the IIFAS provides a reliable measure for better understanding the relationships between breastfeeding knowledge, attitudes, and practices, which is essential for theory-building in breastfeeding education [48]. Additionally, educators can incorporate the IIFAS as an assessment tool to evaluate students’ attitudes toward breastfeeding in academic settings. This can support the development of tailored training programs and help optimize the effectiveness of breastfeeding education across healthcare disciplines.

## 5. Conclusions

The Vietnamese version of the IIFAS is a valuable tool with excellent reliability and validity for assessing breastfeeding attitudes among healthcare students. This tool provides health educators with a reliable means to evaluate students’ attitudes toward breastfeeding and develop targeted educational programs for fostering positive attitudes. By integrating such interventions into healthcare curricula, educators can help prepare students to support breastfeeding mothers effectively, ultimately contributing to improved breastfeeding outcomes. Future studies are warranted to explore the longitudinal impact of breastfeeding education on students’ attitudes as they progress through clinical training and transition to professional practice.

## Figures and Tables

**Table 1:** Sociodemographic characteristics of participants (n = 542).

Variables	Total Sample (n = 542)		EFA Sample (n = 271)		CFA Sample (n = 271)		t/χ^2^	*p*
	*n*	%	*n*	%	*n*	%		
Age (years), mean (SD)	21.0 (1.19)		22.49 (1.07)		22.48 (1.32)		0.11	0.92
GPA, mean (SD)	2.93 (0.33)		2.95 (0.33)		2.91 (0.33)		1.31	0.19
Gender								
Male	142	26.2	79	29.2	63	23.2	0.70	0.48
Female	400	73.8	192	70.8	208	76.8		
Religion								
No religion	392	72.3	188	69.4	204	75.3	2.36	0.13
Buddhist/Catholic	150	27.7	83	30.6	67	24.7		
Ethnicity								
Kinh	462	85.2	232	85.6	230	84.9	0.59	0.81
Khmer/Hoa	80	14.8	39	14.4	41	15.1		
Year of study								
3rd year	98	18.1	45	16.6	53	19.6	0.81	0.67
4th year	221	40.8	112	41.3	109	40.2		
5th year	223	41.1	114	42.1	109	40.2		
Student major								
Medical students	223	41.1	114	42.1	109	40.2	1.88	0.39
Nursing students	280	51.7	134	49.4	146	53.9		
Midwife students	39	7.2	23	8.5	16	5.9		
Ever assisted with BF								
No	98	18.1	45	16.6	53	19.6	0.80	0.37
Yes	444	81.9	226	83.4	218	80.4		

Note: SD, standard deviation; EFA, exploratory factor analysis; CFA, confirmatory factor analysis; GPA, grade point average; BF, breastfeeding.

**Table 2:** Descriptive statistics and internal consistency among exploratory factor analysis sample of the Iowa Infant Feeding Attitude Scale (n = 271).

Items	Mean (SD)	Skewness	Kurtosis	Item-Total Correlation	Alpha if the Item Deleted
1. The nutritional benefits of breast milk last only until the baby is weaned from breast milk.	3.90 (1.06)	−0.94	0.64	0.59	0.93
2. Formula-feeding is more convenient than breast-feeding.	3.97 (1.08)	−1.05	0.63	0.62	0.93
3. Breast-feeding increases mother-infant bonding.	4.01 (1.13)	−1.24	0.97	0.65	0.93
4. Breast milk is lacking in iron.	3.97 (1.09)	−1.07	0.66	0.63	0.93
5. Formula-fed babies are more likely to be overfed than are breast-fed babies.	3.99 (1.05)	−0.96	0.45	0.61	0.93
6. Formula-feeding is the better choice if a mother plans to work outside the home.	3.98 (1.17)	−1.14	0.96	0.68	0.93
7. Mothers who formula-fed are miss out one of the great joys of motherhood.	3.92 (1.11)	−1.07	0.38	0.60	0.93
8. Mothers should not breast-feed in public places such as restaurants.	4.00 (1.10)	−1.10	0.59	0.66	0.93
9. Babies fed breast milk are healthier than babies who are fed formula.	4.01 (1.10)	−1.10	0.63	0.67	0.93
10. Breast-fed babies are more likely to be overfed than are formula-fed babies.	3.99 (1.10)	−1.25	1.06	0.68	0.93
11. Fathers feel left-out if a mother breast feeds.	3.95 (1.02)	−1.08	0.90	0.66	0.93
12. Breast milk is the ideal food for babies.	4.00 (1.04)	−1.10	0.97	0.71	0.93
13. Breast milk is more easily digested than formula.	3.93 (1.11)	−1.01	0.43	0.66	0.93
14. Formula is as healthy for an infant as breast milk.	3.92 (1.09)	−1.15	0.95	0.66	0.93
15. Breast-feeding is more convenient than formula feeding.	4.04 (1.11)	−1.28	1.06	0.70	0.93
16. Breast milk is less expensive than formula.	3.97 (1.09)	−1.02	0.44	0.62	0.93
17. A mother who occasionally drinks alcohol should not breast-feed her baby.	3.92 (1.09)	−1.11	0.77	0.68	0.93

Note: SD, standard deviation.

**Table 3:** Factor structure of the Iowa Infant Feeding Attitude Scale identified by Exploratory Factor Analysis (n = 271).

Items	Eigenvalue	Explained Variance (%)	Factor Loading
Factor 1: Favorable to breastfeeding	4.74	28.00	
16. Breast milk is less expensive than formula.			0.80
17. A mother who occasionally drinks alcohol should not breast-feed her baby.			0.79
3. Breast-feeding increases mother-infant bonding.			0.77
15. Breast-feeding is more convenient than formula feeding.			0.77
13. Breast milk is more easily digested than formula.			0.75
7. Mothers who formula-fed are miss out one of the great joys of motherhood.			0.66
5. Formula-fed babies are more likely to be overfed than are breast-fed babies.			0.68
9. Babies fed breast milk are healthier than babies who are fed formula.			0.64
12. Breast milk is the ideal food for babies.			0.63
Factor 2: Favorable to formula feeding	4.36	26.00	
11. Fathers feel left-out if a mother breast feeds.			0.81
10. Breast-fed babies are more likely to be overfed than are formula-fed babies.			0.80
4. Breast milk is lacking in iron.			0.75
14. Formula is as healthy for an infant as breast milk.			0.73
2. Formula-feeding is more convenient than breast-feeding.			0.70
6. Formula-feeding is the better choice if a mother plans to work outside the home.			0.68
1. The nutritional benefits of breast milk last only until the baby is weaned from breast milk.			0.65
8. Mothers should not breast-feed in public places such as restaurants.			0.64

**Table 4:** Correlations of the Iowa Infant Feeding Attitude Scale total score, factor score with breastfeeding knowledge and anxiety symptoms among healthcare students (n = 271).

Variables	IIFAS Total Score	IIFAS Factors	
		Favorable to Breastfeeding	Favorable to Formula Feeding
BF knowledge	0.74 ***	0.64 ***	0.67 ***
Anxiety symptoms	−0.13 *	−0.16 **	−0.06

Note: IIFAS, Iowa Infant Feeding Attitude Scale; * *p* < 0.05, ** *p* < 0.01, *** *p* < 0.001.

**Table 5:** Association between sociodemographics and breastfeeding attitude of participants (n = 542).

Variables	Breastfeeding Attitude (IIFAS)			
	Mean (SD)	t/F	*p*-Value	Post Hoc Comparison Between Groups
Gender		0.74	0.46 ^a^	
Male	67.51 (12.49)			
Female	66.55 (13.60)			
Religion		1.81	0.07 ^a^	
No religion	67.48 (12.45)			
Buddhist/Catholic	65.18 (15.14)			
Ethnicity		0.17	0.87 ^a^	
Kinh	66.88 (13.29)			
Khmer/Hoa	66.61 (13.26)			
Year of study		4.63	0.01 ^b^	5th > 4th
3rd year	65.52 (14.33)			
4th year	65.35 (14.85)			
5th year	68.90 (10.68)			
Student major		5.68	0.004 ^b^	Medical > Nursing, Medical > Midwife
Medical students	68.90 (10.68)			
Nursing students	65.80 (14.39)			
Midwife students	62.56 (16.45)			
Ever assisted with BF		−1.11	0.27 ^a^	
No	65.50 (14.45)			
Yes	67.14 (13.00)			

Note: SD, standard deviation; IIFAS, Iowa Infant Feeding Attitude Scale, BF, breastfeeding. ^a^ Independent samples *t*-test, ^b^ one-way ANOVA, according to Bonferroni post hoc tests.

## Data Availability

The data are available from the authors upon request.

## Supplementary Materials

The following supporting information can be downloaded at https://www.mdpi.com/article/10.3390/healthcare13111233/s1, Table S1: Factor loadings of the Iowa Infant Feeding Attitude Scale identified by confirmatory factor analysis (n = 271).
