# Supplementary material for: Psychometric Validation of the Iowa Infant Feeding Attitude Scale Among Healthcare Students in Vietnam
Source: Healthcare (Basel). 2025 May 23;13(11):1233. doi: 10.3390/healthcare13111233 (PMC12154217; doi:10.3390/healthcare13111233)
Supplement: Supplementary file 1 [file healthcare-13-01233-s001.zip › healthcare-3625114-supplementary.pdf]

Table S1.

Factor loadings of the Iowa Infant Feeding Attitude Scale identified Confirmatory Factor Analysis (n=271).

Items	Factor loading
Factor 1: Favorable to breastfeeding	
17. A mother who occasionally drinks alcohol should not breast-feed her baby.	0.78
16. Breast milk is less expensive than formula.	0.76
3. Breast-feeding increases mother-infant bonding.	0.75
15. Breast-feeding is more convenient than formula feeding.	0.72
13. Breast milk is more easily digested than formula.	0.72
5. Formula-fed babies are more likely to be overfed than are breast-fed babies.	0.72
9. Babies fed breast milk are healthier than babies who are fed formula.	0.74
12. Breast milk is the ideal food for babies.	0.77
7. Mothers who formula-fed are miss out one of the great joys of motherhood.	0.79
Factor 2: Favorable to formula feeding	
11. Fathers feel left-out if a mother breast feeds.	0.76
10. Breast-fed babies are more likely to be overfed than are formula-fed babies.	0.67
4. Breast milk is lacking in iron.	0.75
14. Formula is as healthy for an infant as breast milk.	0.73
2. Formula-feeding is more convenient than breast-feeding.	0.75
6. Formula-feeding is the better choice if a mother plans to work outside the home.	0.73
1. The nutritional benefits of breast milk last only until the baby is weaned from breast milk.	0.77
8. Mothers should not breast-feed in public places such as restaurants.	0.71
